# Obstructive Sleep Apnea Is Associated With Low Testosterone Levels in Severely Obese Men

**DOI:** 10.3389/fendo.2021.622496

**Published:** 2021-07-26

**Authors:** Milina Tančić-Gajić, Miodrag Vukčević, Miomira Ivović, Ljiljana V. Marina, Zorana Arizanović, Ivan Soldatović, Miloš Stojanović, Aleksandar Đogo, Aleksandra Kendereški, Svetlana Vujović

**Affiliations:** ^1^ Department for Obesity, Reproductive and Metabolic Disorders, Clinic for Endocrinology, Diabetes and Metabolic Diseases, University Clinical Centre of Serbia, Faculty of Medicine, University of Belgrade, Belgrade, Serbia; ^2^ Department of Pulmonology, Clinical Hospital Centre Zemun, Faculty of Medicine, University of Belgrade, Belgrade, Serbia; ^3^ Institute of Medical Statistics and Informatics, Faculty of Medicine, University of Belgrade, Belgrade, Serbia; ^4^ Department of Endocrinology, Clinical Center of Montenegro, Podgorica, Montenegro

**Keywords:** obesity, metabolic syndrome, sleep apnea, testosterone, male

## Abstract

**Background:**

Disrupted sleep affects cardio-metabolic and reproductive health. Obstructive sleep apnea syndrome represents a major complication of obesity and has been associated with gonadal axis activity changes and lower serum testosterone concentration in men. However, there is no consistent opinion on the effect of obstructive sleep apnea on testosterone levels in men.

**Objective:**

The aim of this study was to determine the influence of obstructive sleep apnea on total and free testosterone levels in severely obese men.

**Materials and methods:**

The study included 104 severely obese (Body Mass Index (BMI) ≥ 35 kg/m^2^) men, aged 20 to 60, who underwent anthropometric, blood pressure, fasting plasma glucose, lipid profile, and sex hormone measurements. All participants were subjected to polysomnography. According to apnea-hypopnea index (AHI) patients were divided into 3 groups: <15 (n = 20), 15 - 29.9 (n = 17) and ≥ 30 (n = 67).

**Results:**

There was a significant difference between AHI groups in age (29.1 ± 7.2, 43.2 ± 13.2, 45.2 ± 10.2 years; p < 0.001), BMI (42.8 ± 5.9, 43.2 ± 5.9, 47.1 ± 7.8 kg/m^2^; p = 0.023), the prevalence of metabolic syndrome (MetS) (55%, 82.4%, 83.6%, p = 0.017), continuous metabolic syndrome score (siMS) (4.01 ± 1.21, 3.42 ± 0.80, 3.94 ± 1.81, 4.20 ± 1.07; p = 0.038), total testosterone (TT) (16.6 ± 6.1, 15.2 ± 5.3, 11.3 ± 4.44 nmol/l; p < 0.001) and free testosterone (FT) levels (440.4 ± 160.8, 389.6 ± 162.5, 294.5 ± 107.0 pmol/l; p < 0.001). TT level was in a significant negative correlation with AHI, oxygen desaturation index (ODI), BMI, MetS and siMS. Also, FT was in a significant negative correlation with AHI, ODI, BMI, age, MetS and siMS. The multiple regression analysis revealed that both AHI and ODI were in significant correlation with TT and FT after adjustment for age, BMI, siMS score and MetS components.

**Conclusion:**

Obstructive sleep apnea is associated with low TT and FT levels in severely obese men.

## Introduction

Obesity is a complex metabolic disorder with a markedly increased prevalence in both the developed and underdeveloped countries. Over the past four decades, the percentage of obese people has doubled among females and quadrupled among males (1, 2).

Excess body weight is a crucial risk factor for mortality and morbidity, especially in obese men with body mass index (BMI) over 35 kg/m^2^ (1, 3). The risk of developing male infertility increases with obesity severity. Alterations in sex steroid hormones contribute to infertility in obese men (4). Obesity in men is associated with low testosterone and low measured or calculated free and bioavailable testosterone (1). Men with a BMI of 35–40 kg/m^2^ can have up to 50% less free and total testosterone when compared to age-matched peers with a normal BMI (5). Low testosterone levels in obese men are considered a consequence of reduced sex hormone binding globulin (SHBG) synthesis, increased androgens aromatization to estradiol, and central gonadal axis suppression. Complex metabolic disorders, increased pro-inflammatory adipocytokines, impaired insulin signaling in the central nervous system, dysregulated leptin signaling, and increased estrogen may lead to hypothalamic suppression *via* effects on kisspeptin neurons in obese men (6, 7).

Furthermore, metabolic syndrome, as an adverse health consequence of obesity, is associated with lower testosterone levels independent of age and BMI (8, 9). Disrupted sleep affects cardio-metabolic and reproductive health. The most important clinical cause of disrupted sleep is obstructive sleep apnea syndrome (OSAS) (10). OSAS is emerging as a new area of interest for andrological issues (4). It is characterized by repetitive episodes of upper airway obstruction that occur during sleep and is associated with a complete (apnea) or incomplete (hypopnea) cessation of airflow. This is commonly accompanied by loud snoring and reduction in blood oxygen saturation, followed by arousal, fragmented sleep, and daytime sleepiness (11, 12). The most important epidemiological risk factor for sleep apnea is obesity, preceding age, and male gender (13). The prevalence of OSAS in obese individuals is over 30%, and 50-98% in morbidly obese patients (14). There is growing evidence to support an independent association of OSAS with cardiovascular, neuropsychiatric, pulmonary, and renal disorders as well as with metabolic and endocrine co-morbidities. There is convincing evidence that OSAS is also an independent risk factor for metabolic syndrome (15). It is clinically noticeable that obese men with sleep apnea have lower than expected concentrations of total testosterone (TT) and free testosterone (FT) (3, 9, 10). However, the independent effect of OSAS on blood testosterone concentrations has been shown in some (9, 16–20) but not in all cross-sectional studies (21, 22).

Considering the inconclusive data, the purpose of this study was to determine the influence of obstructive sleep apnea on TT and FT levels in severely obese men.

## Materials and Methods

### Subjects

This was an observational, cross-sectional study. We have evaluated 165 severely obese men (BMI *≥* 35kg/m^2^), aged 20 to 60, admitted to the Department for Obesity, Metabolic and Reproductive Disorders at the Clinic for Endocrinology, Diabetes and Metabolic Diseases University, Clinical Centre of Serbia between 2006 and 2016.

**Figure 1:**
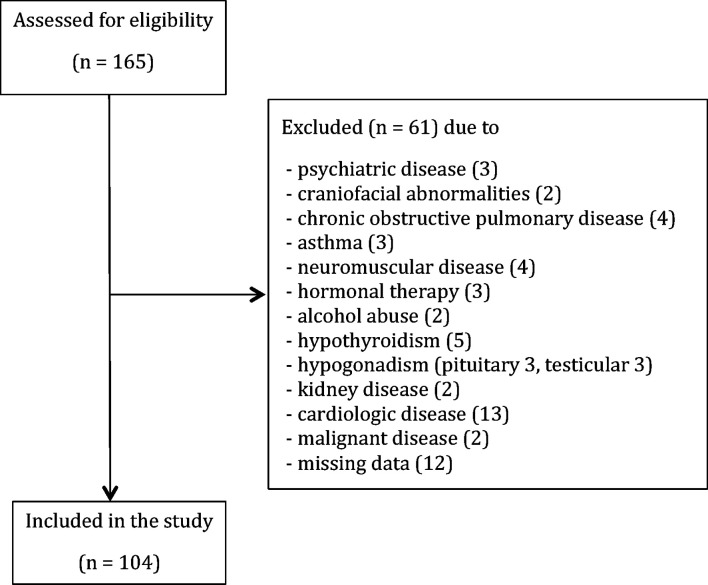
STROBE flowchart of the present study.

Detailed personal history, the biochemical and endocrinological evaluation was conducted in order to detect and exclude patients with: hypercortisolism (0) and/or hypothyroidism (5), history of alcohol consumption (≥ 2 units per day) or substance abuse (2), hormonal therapy (3), hypogonadism due to the pituitary (3) or testicular diseases (3), craniofacial abnormalities (2), liver or kidney diseases (2), neuromuscular diseases (4), chronic obstructive pulmonary disease (4), asthma (3), manifest cardiologic diseases (13), malignancies (2), psychiatric diseases (3) and missing data (12). In total, sixty-one patients were excluded from the study as provided in the STROBE flowchart (**Figure 1**).

This study was designed in agreement with the Declaration of Helsinki and was approved by the local Ethical Committee. The patients gave their informed consent.

### Study Protocol

All study patients underwent anthropometric and blood pressure measurements, biochemical and hormonal analysis, as well as the overnight polysomnography study.

#### Anthropometry and Measurement of Blood Pressure

Weight was measured in the morning, without shoes, in light clothing, using a medical scale with an accuracy of 0.1 kg. Height was measured without shoes, using a stadiometer with an accuracy of 0.1 cm. Waist, hip, and neck circumferences expressed in centimeters were measured using a non-elastic band in the standing position. Waist circumference was measured at the end of the expiration at the midline between the lowest point of the costal arch and the highest point of the iliac crest. Hip circumference was measured at the level of the large trochanter of the femur (23). Neck circumference was measured at the level of the cricothyroid membrane (24). The systolic (SBP) and diastolic (DBP) blood pressure was measured using a standard sphygmomanometer in the sitting position: three values were obtained with a 2-minute time-lapse in between, and the average was recorded (23).

#### Blood Samples

Fasting blood samples were taken to measure glucose, triglyceride (Tg), high-density lipoprotein (HDL), albumin, luteinizing hormone (LH), follicle-stimulating hormones (FSH), estradiol, testosterone, and SHBG in the morning following polysomnography.

#### Sleep Study

The apnea-hypopnea index (AHI) and the oxygen desaturation index (ODI) were derived from nocturnal sleep studies using a seven-channel portable sleep recorder (Stardust II, Respironics, Inc., USA). The system detected apneas and hypopneas by measuring: pressure-based airflow with snoring detection, pulse rate, arterial oxyhemoglobin saturation, chest or abdominal effort, and body position changes. The sleep studies were scored at the hospital by a certified sleep specialist. Apneas were defined as a total cessation of airflow for at least 10s if a respiratory effort was present. Hypopneas were defined as a decrease in nasal pressure signal of ≥ 30% of baseline, which was associated with a ≥ 3% desaturation. The apnea-hypopnea index (AHI) was calculated as the total number of obstructive apneas and hypopneas per hour of sleep. OSAS was classified based on the AHI as follows: mild (≥ 5 and < 15 events/h), moderate (≥ 15 and < 30 events/h), or severe (≥ 30 events/h). The oxygen desaturation index (ODI) was defined as the number of oxygen desaturations ≥3% per hour of sleep (25).

#### Biochemical and Hormonal Assays

The fasting plasma glucose (FPG) levels were measured by the glucose-oxidase method (Beckman). Fasting serum lipid levels (HDL and Tg) were analyzed enzymatically using a commercial kit (Bushranger Mannheim GmbH Diagnostica). The serum LH (The ImmuChem hLH IRMA kit, ICN Biomedicals, Inc., CA, USA, CV 2.4%), FSH (The ImmuChem FSH- CT IRMA kit, ICN Biomedicals, Inc., CV 2.6%), estradiol (ESTR- US- CT Cisbio, Bioassays, CV 2.8%), testosterone (TESTO-CT2, Cisbio International, CV 3.1%), and SHBG (SHBG-RIACT, Cisbio International, France, CV 3.6%) were measured by radioimmunoassay.

#### Calculations

Metabolic syndrome (MetS) was defined by three of five criteria: FPG ≥ 5.6mmol/l or antidiabetic therapy, increased waist circumference equal to or greater than 94 cm, TG ≥ 1.7mmol/l, HDL < 1.0mmol/l or antilipidemic therapy, and blood pressure ≥ 130/85 mmHg or therapy (26).

To evaluate the metabolic syndrome, we used siMS score - continuous metabolic syndrome score for quantification of patients’ metabolic status. siMS score (siMS) was calculated using the following formula: siMS score = 2*Waist/Height + FPG/5.6 + Tg/1.7 + SBP/130—HDL/1.02 (27).

FT was calculated based on TT, SHBG, and albumin with the formula as reported by Vermeulen et al. (28). TT < 11 nmol/l and FT < 220 pmol/l were deemed low (29).

### Statistical Analysis

Results are presented as count (%), mean ± standard deviation, or median (25th-75th percentile) depending on the data type and distribution. Groups were compared with parametric (ANOVA) and nonparametric (Kruskal-Wallis test, Mantel-Haenszel chi-square test for trend) tests. To test the correlation between the variables, Pearson and Spearman’s correlation were used. Multiple linear regression analysis was performed to evaluate the relationship between the dependent variable and independent variables. All p values below 0.05 were considered significant. All data were analyzed using SPSS 20.0 (IBM Corp. Released 2011. IBM SPSS Statistics for Windows, Version 20.0. Armonk, NY: IBM Corp.).

## Results

The general characteristics of the study subjects are summarized in **Table 1**. The mean age was 41.8 ± 11.9 years, the mean BMI was 45.7 ± 7.4 kg/m^2^, and the mean weight was 144.9 ± 24.2 kg. 77.9% of patients had MetS, 48.1% had low concentrations of the TT, and 29.8% had low concentrations of FT. The prevalence of sleep apnea was 96.2%, out of which 15.4% had mild, 16.3% moderate, and 64.5% had severe obstructive sleep apnea.

**Table 1:** General characteristics of the total cohort and AHI groups.

	Total	AHI			p value
		<15 (n=20)	15-29.9 (n=17)	≥30 (n=67)	
**Anthropometry**					
Age (yrs.)	41.8 ± 11.9	29.1 ± 7.2	43.2 ± 13.2	45.2 ± 10.2	<0.001^a^
BMI (kg/m^2^)	45.7 ± 7.4	42.8 ± 5.9	43.2 ± 5.9	47.1 ± 7.8	0.023^a^
Weight (kg)	144.9 ± 24.2	139.3 ± 15.5	136.2 ± 24.2	148.8 ± 28.5	0.116^a^
Waist circumference (cm)	139.1 ± 15.8	130.2 ± 11.0	133.9 ± 12.7	143.0 ± 16.4	0.002^a^
Hip circumference (cm)	134.9 ± 17.4	133.0 ± 11.6	130.7 ± 14.1	136.6 ± 19.4	0.395^a^
Neck circumference (cm)	48.3 ± 3.9	45.1 ± 2.9	47.7 ± 3.3	49.4 ± 3.7	<0.001^a^
SBP (mmHg)	136.8 ± 15.7	130.0 ± 13.6	129.7 ± 11.5	140.6 ± 16.0	0.003^a^
DBP (mmHg)	88.2 ± 12.2	81.2 ± 11.2	86.0 ± 11.7	90.8 ± 11.9	0.005^a^
Hypertension	85 (81.7%)	11 (55.0%)	14 (82.4%)	60 (89.6%)	0.001^c^
**Habits**					
Smoking	47 (45.2%)	11 (55.0%)	6 (35.3%)	30 (44.8%)	0.483^c^
**Biochemistry**					
HDL (mmol/L)	0.99 ± 0.23	0.93 ± 0.22	1.11 ± 0.31	0.99 ± 0.21	0.049^a^
Tg (mmol/L)	2.34 ± 1.55	1.86 ± 1.11	2.47 ± 2.09	2.46 ± 1.51	0.035^b^
FPG (mmol/L)	4.9 (4.5-5.8)	4.5 (3.9-4.9)	4.9 (4.4-5.9)	5.2 (4.6-6.2)	<0.002^b^
T2DM	28 (26.9%)	2 (10.0%)	7 (41.2%)	19 (28.4%)	0.229^c^
**Metabolic syndrome**					
MetS	81 (77.9%)	11 (55.0%)	14 (82.4%)	56 (83.6%)	0.013^c^
MetS No of comp.					
1	2 (1.9%)	1 (5%)	1 (5.9%)	0	0.029^b^
2	21 (20.2%)	8 (40%)	2 (11.8%)	11 (16.4%)
3	26 (25.0%)	4 (20%)	8 (47.1%)	14 (20.9%)
4	36 (34.6%)	5 (25%)	3 (17.6%)	28 (41.8%)
5	19 (18.3%)	2 (10%)	3 (17.6%)	14 (20.9%)
siMS	4.01 ± 1.21	3.42 ± 0.80	3.94 ± 1.81	4.20 ± 1.07	0.038^a^
**Sex hormones**					
FSH (IU/l)	4.9 (3.0-7.8)	4.6 (2.4-7.0)	6.2 (3.9-7.4)	4.9 (3.2-8.3)	0.400^b^
Estradiol (pmol/l)	121.1 ± 51.5	108.6 ± 65.9	104.4 ± 49.9	129.2 ± 45.8	0.114^a^
LH (IU/l)	3.62 ± 1.88	3.57 ± 1.29	4.49 ± 1.67	3.41 ± 2.03	0.105^a^
SHBG (nmol/l)	18.4 (11.9-26.3)	19.3 (12.8-27.6)	16.1 (11.4-32.8)	18.5 (11.9-24.7)	0.860^b^
T (nmol/l)	12.9 ± 5.4	16.6 ± 6.1	15.2 ± 5.3	11.3 ± 4.4	<0.001^a^
FT (pmol/l)	338.1 ± 141.2	440.4 ± 160.8	389.6 ± 162.5	294.5 ± 107.0	<0.001^a^
TT < 11 (nmol/l)	44 (42.3%)	6 (30.0%)	2 (11.8%)	36 (53.7%)	0.012^c^
FT < 220 (pmol/l)	21 (20.2%)	0	2 (11.8%)	19 (28.4%)	0.004^c^
T T< 11(nmol/l) & FT<220 (pmol/l)	20 (19.2%)	0	1 (5.9%)	19 (28.4%)	0.002^c^

AHI, apnea-hypopnea index; BMI, body mass index; SBP, systolic blood pressure; DBP, diastolic blood pressure; FPG, fasting plasma glucose; HDL, high density lipoprotein; Tg, triglycerides; T2DM, type 2 diabetes mellitus; MetS, metabolic syndrome; siMS, continuous metabolic syndrome score; FSH, follicle -stimulating hormone; LH, luteinizing hormone; SHBG, sex hormone-binding globulin; TT, Total Testosterone; FT, Free Testosterone; ^a^ANOVA, ^b^Kruskal-Wallis, ^c^Mantel Haenszel chi-square test for trend.

Patients were divided into groups according to AHI levels in order to present values of clinical parameters in an easy-to-understand fashion. Taking into account that the group with no OSAS was too small (only 4 patients), we classified patients into 3 AHI groups: AHI < 15 (20 patients), AHI 15 - 29.9 (17 patients), and AHI ≥ 30 (67 patients) as presented in **Table 1**. Our results showed a clear significant difference between AHI groups in TT and FT levels (p<0.001). There was also a significant difference between AHI groups in terms of age, BMI, waist and neck circumference, blood pressure levels, hypertension prevalence, triglyceride, high-density lipoprotein and FPG levels, metabolic syndrome prevalence, and siMS score (**Table 1**). There was no significant difference in TT (12.8 ± 5.3 *vs.* 13.1 ± 5.5 nmol/l; p = 0.734) or FT level (346.1 ± 124.9 *vs.* 331.6 ± 154.1 pmol/l; p = 0.605), AHI (46.4 ± 29.7 *vs.* 44.7 ± 27.4; p = 0.762) and ODI (44.5 ± 31.8 *vs.* 42.7 ± 29.9; p = 0.767) between smokers and non-smokers.

**Figure 2:**
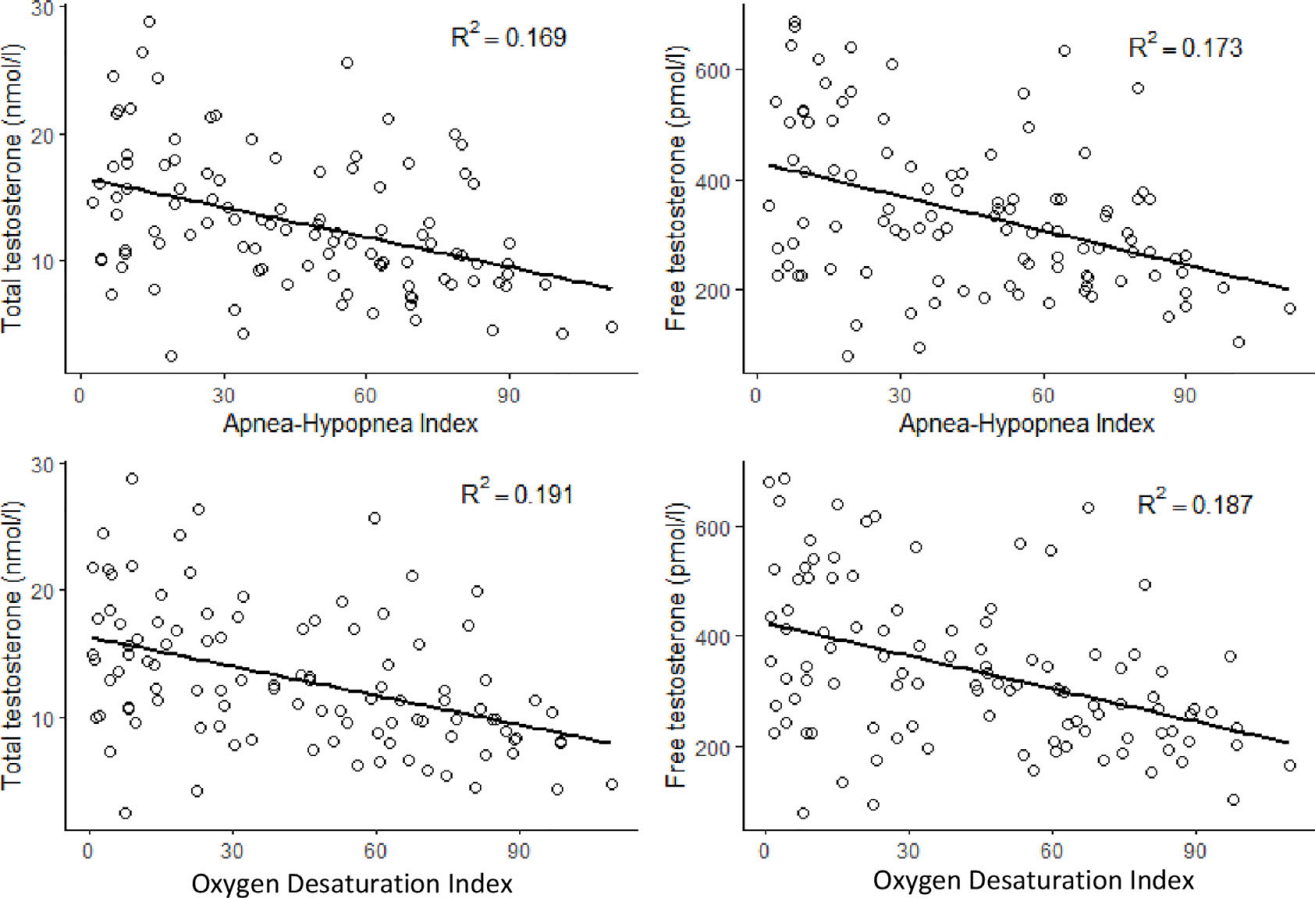
Correlations between total testosterone and free testosterone with apnea-hypopnea index and oxygen desaturation index.

There was a significant negative correlation between TT and AHI (r = -0.409, p < 0.001) and ODI (r = -0.458, p < 0.001) levels. There was also a significant negative correlation between FT and AHI (r = -0.389, p < 0.001) and ODI (r = -0.438, p <0.001) levels (**Figure 2**). Both TT and FT levels were in a significant negative correlation with BMI (r = -0.269, p = 0.006 and r = -0.311, p = 0.001, respectively), weight (r = -0.203, p = 0.039 and r = -0.227, p = 0.02, respectively), hip circumference (r = -0. 234, p = 0.017 and r = -0.243, p = 0.013, respectively), some metabolic parameters such as waist circumference (r = -0.374, p < 0.001 and r = -0.398, p < 0.001, respectively) DBP (r = -0.264, p = 0.007 and r = -0.294, p = 0.002, respectively), FPG (r = -0.274, p = 0.005 and r = -0.296, p = 0.002, respectively), and also with MetS prevalence r = -0.193, p = 0.049 and r = -0.195, p = 0.048, respectively) and siMS score levels (r = -0.321, p = 0.001 and r = -0.283, p = 0.004, respectively) (**Table 2**). FT levels were in a significant negative correlation with age (r = -0.346, p<0.001) (**Table 2**).

**Table 2:** Correlation matrix between testosterone, free testosterone, AHI, ODI and general characteristics of patients.

	TT^a^	FT^a^	AHI^b^	ODI^b^
TT	1	.847^**^	-.409^**^	-.458^**^
FT	.847^**^	1	-.389^**^	-.438^**^
AHI	-.412^**^	-.416^**^	1.000	.916^**^
ODI	-.437^**^	-.433^**^	.916^**^	1.000
Age	-.170	-.346^**^	.320^**^	.339^**^
BMI	-.269^**^	-.311^**^	.382^**^	.391^**^
Waist	-.374^**^	-.398^**^	.413^**^	.429^**^
Weight	-.203^*^	-.227^*^	.300^**^	.298^**^
Neck	-.174	-.225^*^	.490^**^	.469^**^
Hip	-.234^*^	-.243^*^	.230	.246
SBP	-.104	-.166	.280^**^	.324^**^
DBP	-.264^**^	-.294^**^	.314^**^	.322^**^
HDL	.119	.062	.015	-.040
Tg	-.195^*^	-.134	.151	.169
FPG	-.274^**^	-.296^**^	.275^**^	.250^*^
MetS	-.193^*^	-.195^*^	.163	.224^*^
siMS	-.321^**^	-.283^**^	.283^**^	.312^**^
FSH	.060	-.095	.063	.116
LH	.230^*^	.181	-.151	-.093
E	-.070	.016	.210^*^	.174
SHBG	.366^**^	-.122	-.080	-.098

TT, total testosterone; FT, free testosterone; AHI, apnea-hypopnea index ;ODI, oxygen desaturation index; BMI, body mass index; SBP, systolic blood pressure; DBP, diastolic blood pressure; HDL, high density lipoprotein; Tg, triglycerides; FPG, fasting plasma glucose; MetS, metabolic syndrome; siMS, continuous metabolic syndrome score; FSH, follicle -stimulating hormone; LH, luteinizing hormone; Results are presented as Pearson correlation coefficient or Spearman`s rank correlation coefficient, ^a^Pearson correlation; ^b^Spearman correlation; *p < 0.05 **p < 0.01

Furthermore, there was a significant positive correlation between AHI and ODI levels with age (r = 0.320, p = 0.001 and r = 0.339, p < 0.001, respectively), BMI (r = 0.382, p < 0.001 and r = 0.391, p < 0.001, respectively), weight (r = 0.300, p = 0.002 and r = 0.298, p = 0.002, respectively) waist (r = 0.413, p < 0.001 and r = 0.429, p < 0.001, respectively), hip (r = 0.230, p = 0.019 and r = 0.246, p = 0.012) and neck circumference (r = 0.490, p < 0.001 and r = 0.469, p < 0.001, respectively), SBP (r = 0.280, p = 0.004 and r = 0.324, p = 0.001, respectively), DBP (r = 0.314, p = 0.001 and r = 0.322, p = 0.001, respectively), FPG (r = 0.275, p = 0.005 and r = 0.250, p = 0.001, respectively), and siMS score levels (r = 0.283, p = 0.004 and r = 0.312, p< 0.001, respectively) (**Table 2**).

In multiple regression analysis, after adjustment for age, BMI, siMS score and MetS components, both AHI and ODI were in significant correlation with TT and FT (p < 0.05) (**Table 3**). The results of multiple linear regression analysis did not change in subgroup of patients with BMI ≥ 40kg/m^2^ (80 patients in total) (**Table 4**). The calculated variance inflation factors showed no multicollinearity in regression models.

**Table 3:** Multiple linear regression model for prediction of total testosterone (TT) and free testosterone (FT).

	TT		FT	
	B (95% CI)	Adj R^2^	B (95% CI)	Adj R^2^
AHI	-0.078 (-0.112 to -0.044)	0.162	-2.071 (-2.961 to -1.182)	0.165
AHI adjusted for age, BMI, siMS score	-0.060 (-0.098 to -0.022)	0.216	-1.088 (-2.057 to -0.120)	0.273
AHI adjusted for age, waist circumference, SBP, FPG, Tg, HDL	-0.059 (-0.096 to -0.021)	0.232	-1.185 (-2.145 to -0.226)	0.266
ODI	-0.077 (-0.108 to -0.046)	0.183	-1.991 (-2.804 to -1.177)	0.180
ODI adjusted for age, BMI, siMS score	-0.060 (-0.096 to -0.025)	0.229	-1.070 (-1.970 to -0.170)	0.277
ODI adjusted for age, waist circumference, SBP, FPG, Tg, HDL	-0.061 (-0.096 to -0.027)	0.251	-1.208 (-2.104 to -0.313)	0.274

p values in all models are < 0.05, AHI, apnea-hypopnea index; BMI, body mass index; siMS, continuous metabolic syndrome score; SBP, systolic blood pressure; FPG, fasting plasma glucose; Tg, triglycerides; HDL, high density lipoprotein; ODI, oxygen desaturation index.

**Table 4:** Multiple linear regression model for prediction of total testosterone (TT) and free testosterone (FT) for patients with BMI ≥ 40 kg/m^2^.

	TT		FT	
	B (95% CI)	Adj R^2^	B (95% CI)	Adj R^2^
AHI	-0.070 (-0.109 to -0.031)	0.129	-1.995 (-2.971 to -1.019)	0.164
AHI adjusted for age, BMI, siMS score	-0.071 (-0.112 to -0.029)	0.267	-1.511 (-2.572 to -0.450)	0.257
ODI	-0.068 (-0.103 to -0.033)	0.148	-1.832 (-2.722 to -1.941)	0.166
ODI adjusted for age, BMI, siMS score	-0.069 (-0.106 to -0.032)	0.284	-1.379 (-2.341 to -0.417)	0.258

p values in all models are < 0.05, AHI, apnea-hypopnea index; BMI, body mass index; siMS, continuous metabolic syndrome score; ODI, oxygen desaturation index.

**Figure 3:**
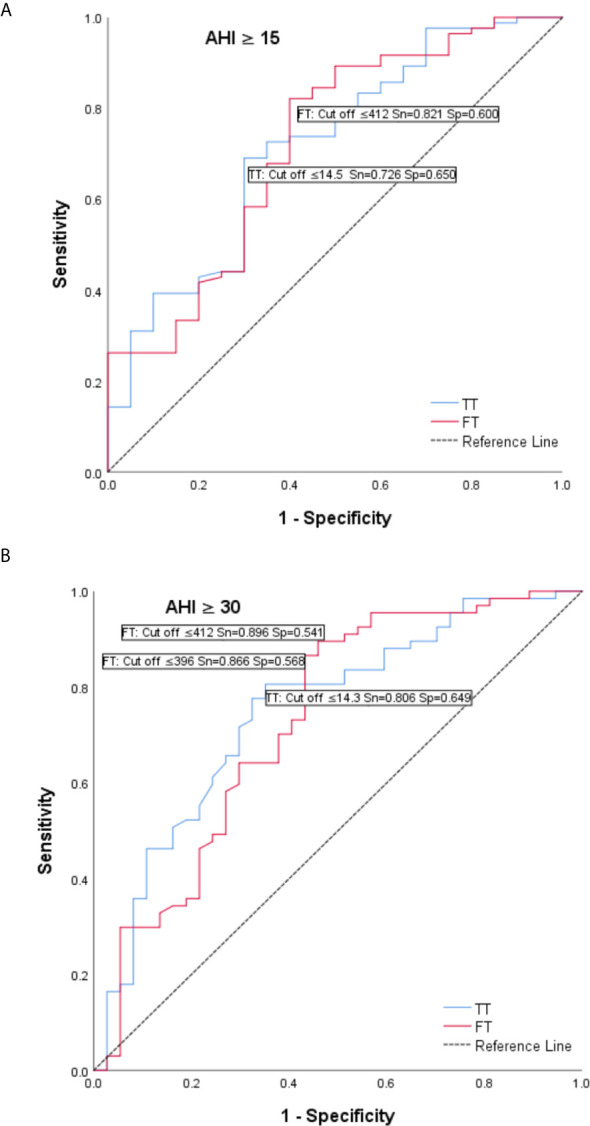
Receiver operating characteristic (ROC) curves – total testosterone (T) and tree testosterone (FT) as discriminative variables for the assessment of sleep apnea severity. **(A)** AUC for AHI ≥ 15 for TT was AUC_T_ = 0.714 (95% CI 0.587 - 0.841; p = 0.003) with cut off = 14.5 (Sn = 0.726; Sp = 0.650), and for FT AUC_FT_ = 0.719 (95% CI 0.588 - 0.851; p = 0.002) with cut off = 412 (Sn = 0.821; Sp = 0.600). **(B)** AUC for AHI ≥ 30 for TT was AUC_T_ = 0.748 (95% CI 0.648 - 0.849; p < 0.001) with cut off = 14.3 (Sn = 0.806; Sp = 0.649) and for FT AUC_FT_ = 0.728 (95% CI 0.620 - 0.836; p < 0.001) with cut off = 396 (Sn = 0.866; Sp = 0.568).

In order to assess if TT and FT can be used as discriminative variables for the assessment of sleep apnea severity (using 15 and 30 level cut-off for AHI) we have performed receiver-operating characteristic (ROC) curves analysis. AUC for AHI ≥ 15 for TT was AUC_TT_ = 0.714 (95% CI 0.587 - 0.841; p = 0.003) with cut off = 14.5 (Sn = 0.726; Sp = 0.650), and for FT AUC_FT_ = 0.719 (95% CI 0.588 - 0.851; p = 0.002) with cut off = 412 (Sn = 0.821; Sp = 0.600) (**Figure 3A**).

AUC for AHI ≥ 30 for TT was AUC_TT_ = 0.748 (95% CI 0.648 - 0.849; p < 0.001) with cut off = 14.3 (Sn = 0.806; Sp = 0.649) and for FT AUC_FT_ = 0.728 (95% CI 0.620 - 0.836; p < 0.001) with cut off = 396 (Sn = 0.866; Sp = 0.568) (**Figure 3B**).

## Discussion

Obesity is considered to be the main cause of hypogonadism in men. Hypogonadism is a clinical entity characterized by low serum TT concentration and/or FT concentration and associated symptoms and signs of testosterone deficiency (30). Obesity-related hypogonadism is functional, and it can be reverted by substantial weight loss achieved with non-surgical or surgical interventions (31). Hypoandrogenemia is a term referring to the finding of subnormal testosterone concentrations in men without taking into consideration clinical symptoms or signs of decreased serum testosterone levels. The prevalence of hypoandrogenemia is 4% to 5% in the general male population and as much as 20% to 40% in obese men (30). In our study, 48.1% of severely obese men had TT level < 348.3 ng/dl, and 29.8% had FT level < 70.0 pg/ml. Our results are consistent with the previous data that the presence of low TT and FT in men is closely related to the increased BMI with the highest hypoandrogenemia in more severe obesity (30).

Age is the most prominent predictor of most diseases in humans. Chronological aging per se and age-related changes in overall health and lifestyle are associated with natural declines in serum testosterone (32), presented in our study as a negative relationship between FT and age. Abdominal adiposity is one of five clinical risk factors used as diagnostic criteria for metabolic syndrome. Although obesity and metabolic syndrome frequently coexist, this is not always the case.

A significant proportion of obese individuals do not have metabolic syndrome, and, conversely, metabolic syndrome may be present in non-obese individuals (33). In our study, 77.9% of severely obese men had metabolic syndrome. The metabolic syndrome in obese men is associated with a further decline in testosterone level, with a negative inverse relationship between TT and/or FT levels and metabolic syndrome (8, 34, 35), also observed in our study.

The most striking result to emerge from our data is that OSAS, measured by both AHI and ODI, is an independent determinant of serum testosterone concentration in severely obese men after adjustment for BMI, age, or siMS score. Moreover, the ROC analysis showed that both TT and FT levels could be used as discriminative variables for the assessment of sleep apnea severity.

Patients with OSAS have reduced quality and quantity of sleep due to sleep fragmentation, intermittent nocturnal hypoxia, reduced deep and rapid eye movement (REM) sleep, reduced sleep duration, and sleep efficiency, all of which lead to pituitary-gonadal dysfunction and low testosterone levels in male patients (5, 36).

The association of respiratory hypoxia and low serum testosterone in men was observed as early as 40 years ago when it was shown that in patients with chronic obstructive pulmonary disease, erectile dysfunction and low serum testosterone concentrations correlated with the degree of arterial hypoxia (37, 38). A couple of years later, the same results were obtained in patients with pulmonary fibrosis (39), pulmonary heart (40), and Pickwick syndrome (41). Kouchiyama et al., in the study conducted on 24 patients, showed that greater nocturnal oxygen desaturation in men led to the disruption of the circadian rhythm of testosterone secretion (42).

In the animal models, studies focusing on the effects of continuous or intermittent hypoxia on sex hormones were inconclusive because - depending on the study - they showed an increase (43), decrease (44, 45), and unchanged testosterone concentrations (46).

Partial or complete upper airway obstruction in OSAS patients is the cause of not only nocturnal oxygen desaturation but also sleep fragmentation. A significant number of studies have been carried out by Luboshitzky and colleagues to investigate whether reproductive hormones are correlated with sleep patterns in men with OSAS. They showed that the patients with fragmented sleep had a blunted nocturnal rise of testosterone only if they did not show REM sleep (47). The same team suggested that OSAS in male patients is associated with reduced androgen secretion resulting from altered pituitary-gonadal function (48). This is caused by obesity and aging, with hypoxia and sleep fragmentation being additional contributing factors in decreasing pulsatile testosterone secretion in these patients (49).

Clinical evidence is contradictory, with some (9, 16–20), but not all (21, 22), studies reporting that OSAS is a factor favoring hypoandrogenemia independent of obesity. This could be due to high heterogeneity of study design, patients’ characteristics such as age and BMI, exclusion or inclusion criteria, limited or absent covariates in the data analyses, and the time point of data collection (9, 16–22).

Hammoud et al. published a study similar to ours. This study included 89 severely obese men with a BMI ≥ 35 kg/m^2^ to examine the effect of sleep apnea on the reproductive hormones and sexual function in obese men. They showed that increased severity of sleep apnea is associated with lower TT and FT levels independent of age and BMI, which is consistent with the results of our study (18). Their study, unlike our study, did not take into account the metabolic syndrome as a contributing factor to hypoandrogenemia in obese men.

Concerning this latter aspect, there is only one study published by Gambineri et al. that analyzed the severity of OSAS, testosterone levels, and some of the parameters of MetS (waist circumference, FPG, HDL, Tg) in severely obese men. They suggested that OSAS may contribute to causing metabolic abnormalities in men and that this relationship may be in part related to the reduced testosterone concentrations (9). Taking this into consideration, we included metabolic syndrome as a potential confounding factor for hypoandrogenemia in our obese men.

Gambineri and colleagues documented that in men with obesity and OSAS, the severity of hypoxia measured by ODI may be an additional factor in reducing testosterone levels, regardless of BMI and abdominal fatness (9). Our study shows that AHI and ODI, as pivotal markers of OSAS severity are in significant correlation with TT and FT independent of age, BMI, siMS score and MetS components in severely obese men.

The effects of OSAS treatment on testosterone levels are debatable. Continuous positive airway pressure (CPAP) is the most effective non-surgical treatment for OSAS. Furthermore, the efficacy of CPAP on hypoandrogenemia in OSAS male subjects are still controversial. Some studies have demonstrated that CPAP elevates testosterone levels (49, 50). However, the majority of other studies, including two meta-analyses, have reached a different conclusion (51, 52). As pointed out by one of the latest systematic reviews, a small number of included studies in their meta-analysis reported an adequate CPAP use (4 h per night on at least 70% of nights), and thus the results might reflect, at least in part, suboptimal CPAP therapy. Furthermore, Santamaria et al. thoroughly conducted a prospective study of uvulopalatopharyngoplasty therapy effects on testosterone levels in male subjects with moderate and severe OSAS. Interestingly, they showed improvement in testosterone levels three months after the surgery, with correlated improvement in sleep-disordered breathing without significant changes in BMI (53). On the other hand, CPAP as the mainstay of treatment for OSAS will not cure obesity, as a cornerstone of OSAS and metabolic syndrome, as well as hypoandrogenemia in obese men. In any case, significant weight loss clearly improved OSAS, metabolic syndrome, and hypogonadism associated with obesity (1–5, 54).

Some limitations of this investigation should be acknowledged. The design of our study did not provide for monitoring the symptoms of sexual dysfunction and hypogonadism in the subjects. Thus, we can discuss sex hormones only from the perspective of hypoadrogenism and not in terms of hypogonadism. Another limitation of our study is that instead of using ‘full’ polysomnography, we used a seven-channel portable sleep recorder. Given the fact that there was no electroencephalography monitoring, we do not have data about the relationships between sleep stages and sex hormone levels. Also, we could not assess if the physical activity or regular alcohol consumption influenced the results of our study, as we did not have this data available.

## Conclusion

Our data show that obstructive sleep apnea syndrome is in significant correlation with TT and FT levels in severely obese men. Further research is needed to elucidate the complex link between sleep apnea and testosterone levels in obese men for the purpose of appropriate management of these patients.

## Data Availability Statement

The raw data supporting the conclusions of this article will be made available by the authors, without undue reservation.

## Ethics Statement

The studies involving human participants were reviewed and approved by The Faculty of Medicine, University of Belgrade, Ethics Committee. The patients/participants provided their written informed consent to participate in this study.

## Author Contributions

MT-G, MV, and SV: conceived and designed the study, collected and contributed to the data, and analyzed and interpreted data. MT-G: wrote the manuscript. MV and SV: revised the article. MI, LM, ZA, MS, and AĐ: collected and contributed to the data and revised the article. AK: analyzed and interpreted data, and revised the article. IS: analyzed and interpreted data, made tables, and revised the article. All authors contributed to the article and approved the submitted version.

## Conflict of Interest

The authors declare that the research was conducted in the absence of any commercial or financial relationships that could be construed as a potential conflict of interest.

## Publisher’s Note

All claims expressed in this article are solely those of the authors and do not necessarily represent those of their affiliated organizations, or those of the publisher, the editors and the reviewers. Any product that may be evaluated in this article, or claim that may be made by its manufacturer, is not guaranteed or endorsed by the publisher.

## Abbreviations

BMI, body mass index; SHBG, sex hormone-binding globulin; OSAS, obstructive sleep apnea syndrome; Tg, triglyceride; HDL, high-density lipoprotein; LH, luteinizing hormone; FSH, follicle-stimulating hormone; AHI, apnea-hypopnea index; ODI, oxygen desaturation index; MetS, metabolic syndrome; siMS, continuous metabolic syndrome score.
